# Genome-Wide Transcriptional Response of *Mycobacterium smegmatis* MC^2^155 to G-Quadruplex Ligands BRACO-19 and TMPyP4

**DOI:** 10.3389/fmicb.2022.817024

**Published:** 2022-03-04

**Authors:** Egor Shitikov, Dmitry Bespiatykh, Maja Malakhova, Julia Bespyatykh, Ivan Bodoev, Tatiana Vedekhina, Marina Zaychikova, Vladimir Veselovsky, Ksenia Klimina, Elena Ilina, Anna Varizhuk

**Affiliations:** Federal Research and Clinical Center of Physical-Chemical Medicine of Federal Medical Biological Agency, Moscow, Russia

**Keywords:** mycobacteria, transcriptome, RNA, G4, ligands, BRACO-19, TMPyP4

## Abstract

G-quadruplexes (G4s) are non-canonical DNA structures that could be considered as potential therapeutic targets for antimicrobial compounds, also known as G4-stabilizing ligands. While some of these ligands are shown *in vitro* to have a stabilizing effect, the precise mechanism of antibacterial action has not been fully investigated. Here, we employed genome-wide RNA-sequencing to analyze the response of *Mycobacterium smegmatis* to inhibitory concentrations of BRACO-19 and TMPyP4 G4 ligands. The expression profile changed (FDR < 0.05, log_2_FC > |1|) for 822 (515↑; 307↓) genes in *M. smegmatis* in response to BRACO-19 and for 680 (339↑; 341↓) genes in response to TMPyP4. However, the analysis revealed no significant ligand-induced changes in the expression levels of G4-harboring genes, genes under G4-harboring promoters, or intergenic regions located on mRNA-like or template strands. Meanwhile, for the BRACO-19 ligand, we found significant changes in the replication and repair system genes, as well as in iron metabolism genes which is, undoubtedly, evidence of the induced stress. For the TMPyP4 compound, substantial changes were found in transcription factors and the arginine biosynthesis system, which may indicate multiple biological targets for this compound.

## Introduction

The emergence and rapid dissemination of drug-resistant bacterial pathogens are becoming a global health challenge. In 2019, an estimated 10 million people worldwide developed tuberculosis (TB), caused by *Mycobacterium tuberculosis* complex bacteria. In total, nearly half a million people have developed rifampicin-resistant TB (RR-TB), 78% of whom had multidrug-resistant TB (MDR-TB; resistance to both first-line drugs rifampin and isoniazid). The global treatment success rate for MDR/RR-TB was estimated at 57%, compared to an overall success rate of 85% for all cases. Moreover, the treatment of people with resistant TB forms takes longer and requires more expensive and toxic drugs (Seung et al., 2015; Global Tuberculosis Report 2020, 2020). All of these factors encourage the development of new anti-TB agents with new mechanisms of action.

One of the recently emerged trends in the design of antimicrobial drugs suggests targeting quadruplex structures (G4s) in guanine-rich regions of the bacterial genome (Yadav et al., 2021). Such structures comprise planar arrangements of guanine tetrads held together by Hoogsteen base pairing and π–π interactions (Spiegel et al., 2020). G4s have been found in eukaryotes, viruses, and multiple bacterial species (Holder and Hartig, 2014; Ding et al., 2018; Bartas et al., 2019; Marsico et al., 2019), including *M. tuberculosis* (Perrone et al., 2017). Their conservation and non-random genomic distribution (Bartas et al., 2019) are likely indicative of important biological functions. In bacteria, G4s are often found in virulence-related genes (Harris and Merrick, 2015) or their promoter regions and provide a control mechanism involved in the regulation of biological pathways such as transcription and translation (Holder and Hartig, 2014; Wu et al., 2015; Shao et al., 2020). Due to the activity of specific helicases, genomic G4s do not normally persist in cells (Sauer and Paeschke, 2017; Saha et al., 2019). Some G4-resolving helicases (e.g., the PIF1 family) are conserved from bacteria to humans, highlighting the importance of efficient G4 processing (Byrd and Raney, 2017). Unresolved G4s may induce DNA breaks and damage response, recruit transcription factors, or mechanically hamper polymerase passage, affecting transcription and replication (Saranathan and Vivekanandan, 2019).

All the foregoing advocates the use of G4-stabilizing ligands to repress or fine-tune gene expression. An extensive effort is being devoted to designing new small-molecule G4 binders and elucidating the basis for antimicrobial properties of the known ones (Yadav et al., 2021). However, despite recent advances in controlling bacterial growth with exogenous ligands, the underlying mechanisms are poorly understood. Recently, it has been reported that two known G4 ligands, BRACO-19 and c-exNDI2, exhibit anti-TB activity (Perrone et al., 2017); besides, their possible genomic targets have been identified using general sequence rules and G4 prediction algorithms of relatively low stringency (Beaudoin et al., 2014). Subsequently, the stabilizing activity of TMPyP4 ligand for virulence-related G4 targets has been reported (Mishra et al., 2019). These reports encourage the future development of G4-affecting agents. However, additional studies are needed to verify the role of G4s and determine the affected metabolic pathways.

In the present study, we performed the transcriptomic analysis of the *Mycobacterium smegmatis* MC^2^155 strain in the presence of an inhibitory concentration of two G4 ligands, namely BRACO-19 and TMPyP4. Given the known ability of G4 ligands to stabilize G4s, we have identified putative G4 quadruplex sequences in *M. smegmatis* genome and analyzed G4-associated genes during the transcriptomic response to G4 ligands. In addition, we examined genes involved in replication and reparation, with a particular focus on helicases that may be involved in the unwinding of G4-quadruplexes.

## Materials and Methods

### Bacterial Strain, Growth Conditions, and Inhibition Assay

*Mycobacterium smegmatis* MC^2^155 strain was used in this study. Middlebrook 7H11 Agar (HiMedia, India) and Middlebrook 7H9 broth (HiMedia) both supplemented with 0.5% glycerol and 10% Middelbrook OADC Growth Supplement (HiMedia) were used as solid and liquid media, respectively. A frozen stock of *M. smegmatis* strain was cultured on a solid media for 1 day to obtain a sufficient number of cells for inoculation of broth culture.

To determine MICs for BRACO-19 (PubChem CID no. 9808666) and TMPyP4 (PubChem CID no. 135398505; Sigma-Aldrich, United States), bacterial cells were cultured overnight in Middlebrook 7H9 broth, with the subsequent dilution in the fresh 7H9 medium (1:200). The diluted culture was dispensed (196 μl) to the wells of the CELLSTAR 96 Well Cell Culture Plates (Greiner Bio-One GmbH, Germany) and 4 μl of serial 2-fold dilutions of the tested compounds in DMSO were added to the wells to final concentrations of 2.5–80 μM BRACO-19 and 0.25–16 μM for TMPyP4. The plates were incubated for 64 h at 37°C in a humid atmosphere with 5% CO_2_ and shaken at 250 rpm. The MICs were determined by an optical density measurement at 570 nm on xMark Microplate Absorbance Spectrophotometer (Biorad, United States) and defined as the lowest concentration of the compound at which no growth was observed. Negative control samples were treated with the same volume of DMSO; the experiments were performed in three biological replicates.

For the transcriptomic analysis, *M. smegmatis* cells were grown up to the mid-exponential phase (OD_570_ = 0.47) and transferred to the 5 ml tubes (NUOVA APTACA, Italy). Ligands were added to the tubes to a final concentration corresponding to 1 × MIC (10 μM for BRACO-19 and 4 μM for TMPyP4). The same volume of DMSO was added to the control samples (1% v/v). Bacterial cells were incubated for 4 h (cell division time; Logsdon and Aldridge, 2018) at 37°C in a humid atmosphere with 5% CO_2_ and shaken at 250 rpm. The experiments were carried in three biological replicates.

### Total RNA Extraction and RNA-seq

Bacterial cells were harvested by centrifugation (8,000 × *g*, 10 min, 4°C) and subsequently washed twice with a phosphate-buffered saline (10 ml). A double volume of RNAprotect Bacteria Reagent (Qiagen, United States) was added to the pellet. The mixture was incubated at room temperature for 5 min and then centrifuged (8,000 × *g*, 10 min, 4°C). Samples were homogenized for 30 s using an automatic bead homogenizer (MagNAlyzer, Roche). RNA was extracted using the MagMAX mirVana Total RNA Isolation Kit (Thermo Fisher Scientific, Lithuania) on the KingFisher Flex Purification System (Thermo Fisher Scientific, United States) according to the manufacturer’s instructions. RNA was treated with DNase using Turbo DNA-Free Kit (Thermo Fisher Scientific) in the 50 μl volume, and further purified using Agencourt RNAClean XP (Beckman Coulter, United States) according to the manufacturer’s instructions. The total amount of RNA was measured with the Quant-iT Ribogreen RNA assay kit (ThermoFisher Scientific), and the quality of extracted RNA was checked by Agilent Bioanalyzer on Agilent RNA 6000 Pico Chips (Agilent Technologies, United States).

Total RNA (300 ng) was used for library preparation. The Ribo-Zero Plus rRNA Depletion Kit (Illumina, United States) was used to remove rRNA, and the NEBNext Ultra II Directional RNA Library Prep Kit (NEB) was used for library preparation. RNA cleanup was performed with the Agencourt RNA Clean XP kit (Beckman Coulter). Final cleanup was done using the Agencourt AMPure XP system (Beckman Coulter) after which the libraries’ size distribution and quality were assessed using a high sensitivity DNA chip (Agilent Technologies). Libraries were subsequently quantified with Quant-iT High Sensitivity dsDNA Assay Kit (Thermo Fisher Scientific). The libraries were sequenced on an Illumina HiSeq 2500 (50 bp single-end reads), with 12 pM loading concentration, and the PhiX control library (Illumina) spiked in at 5%. The RNA-seq data generated in this study have been deposited to the NCBI Sequence Read Archive under accession number PRJNA765512.^1^

### Inferring Putative G4 Quadruplex Sequences

To infer PQSs in *M. smegmatis* MC^2^155 (GenBank accession no. CP000480.1) genome G4-iM Grinder (v1.6.1; Belmonte-Reche and Morales, 2020) package for R (v4.0.5; R Core Team, 2021) was used. The following parameters were used: Complementary = TRUE, BulgeSize = 0, RunComposition = “G,” MaxRunSize = 4, MinRunSize = 3, MaxNRuns = 0, MinNRuns = 4, MaxIL = 0, MaxPQSSize = 50, MinPQSSize = 15, MaxLoopSize = 15, MinLoopSize = 0. Size independent quadruplexes without overlapping (method 3a) with a score ≥ 40 were used for further analysis. BEDtools (v2.30.0; Quinlan and Hall, 2010) and custom Python scripts were used to assign PQSs to *M. smegmatis* MC^2^155 genes and intergenic regions (Supplementary Table S1). When PQS was located in both gene and intergenic region, it was assigned to both categories. Intergenic PQSs were assigned without regard to strand orientation. For the analysis, only PQSs located upstream (downstream for the template strand) of genes were used. To assign PQSs to promoter regions, a list of previously published *M. smegmatis* MC^2^155 transcription start sites (TSSs) was used (Li et al., 2017). Promoters were considered to be located 50 bp upstream or downstream of TSSs, depending on the strandedness of the gene.

### Circular Dichroism Spectroscopy and Melting Assay

Oligodeoxyribonucleotides (ODNs) were obtained from Litekh, Russia. To verify G4 folding, circular dichroism (CD) spectra of the ODN samples in 10 mM potassium-phosphate buffer, pH 7, were registered at 15°C using Chirascan spectrophotometer (Applied Biophysics, United Kingdom). The ODN samples were annealed rapidly (heated to 90°C and then snap-cooled on ice) to facilitate intramolecular folding prior to all measurements. Moderate salt concentration was used to avoid extremely high thermal stability of the high-scoring G4s, which would hamper accurate assessment of ligands’ effects. Thermal stability (*T*_1/2_) of each G4 was calculated as an average value of the melting (*T*_m_) and annealing (*T*_a_) temperatures estimated based on the annealing and melting curves, respectively. The melting and annealing curves were registered using Chirascan spectrophotometer by CD monitoring at G4-specific maxima (260 nm for parallel-stranded/mixed and 295 nm for antiparallel-stranded/mixed G4s) with a temperature ramp rate of 1°C/min in the presence and in the absence of the ligands. G4 and ligand concentrations were 2.5 μM.

### Differential Gene Expression Analysis

The sequenced reads were mapped to the reference *M. smegmatis* MC^2^155 genome (GenBank accession no. CP000480.1) using HISAT2 (v2.2.1; Kim et al., 2015). SAMtools (v1.11; Li et al., 2009) software was used to sort and convert SAM files to BAM, and their subsequent indexing. Mapping quality and coverage along genes were assessed with QualiMap (v2.2.2; Okonechnikov et al., 2016), individual reports were merged with MultiQC (v1.9; Ewels et al., 2016). Mapped reads were assigned to genes with featureCounts (v2.0.1; Liao et al., 2014). Differential gene expression analysis was performed using the edgeR (v3.30.3; Robinson et al., 2010) package for R. Genes with a false discovery rate (FDR) cutoff of 0.05 and with a fold change (log_2_FC) threshold of |1| (i.e., ≥ |2|-fold change) were considered to be differentially expressed. For intersample comparison, counts were normalized using gene length corrected trimmed mean of M-values (GeTMM; Smid et al., 2018). Further functional enrichment analysis of the GO terms and KEGG pathways for differentially expressed genes was performed using clusterProfiler (v3.18.1; Yu et al., 2012) package, categories were considered enriched with *p*_adj._ ≤ 0.05. In addition, differentially expressed genes were classified into functional categories based on the PATRIC database (Wattam et al., 2014). Clusters of orthologous genes (COG) were annotated by mapping *M. smegmatis* MC^2^155 proteome to NCBI COG database (v2020)^2^ using DIAMOND (v2.0.9; Buchfink et al., 2014), and with eggNOG-mapper (v2; Huerta-Cepas et al., 2019). Plots were generated within R using ggplot2 (v3.3.2; Wickham, 2009), ggforce (v0.3.3; Pedersen, 2021), ggvenn (v0.1.9; Yan, 2021), ggsignif (v0.6.1; Ahlmann-Eltze and Patil, 2021), lemon (v0.4.5; Edwards, 2020), and cowplot (v1.1.0; Wilke, 2020) packages.

### Statistical Analysis

All statistical analyses were performed using RStudio (v1.4.1106). Statistical evaluation was performed by the two-sided Wilcoxon rank-sum test and the Student’s *t*-test; *value of p* < 0.05 was considered to be statistically significant.

## Results

### Differential Gene Expression of *Mycobacterium smegmatis* MC^2^155 in the Presence of BRACO-19 and TMPyP4 G4 Ligands

To understand how G4-stabilizing compounds affect gene expression levels, the transcriptomic profiles of *M. smegmatis* MC^2^155 strain exposed to BRACO-19 and TMPyP4 for 4 h at 1 × MIC were examined (Supplementary Table S1). According to dilution-based antimicrobial susceptibility testing, BRACO-19 and TMPyP4 had *in vitro* minimum inhibitory concentration (MIC) values of 10 and 4 μM, respectively (Supplementary Figure S1).

**Figure 1:**
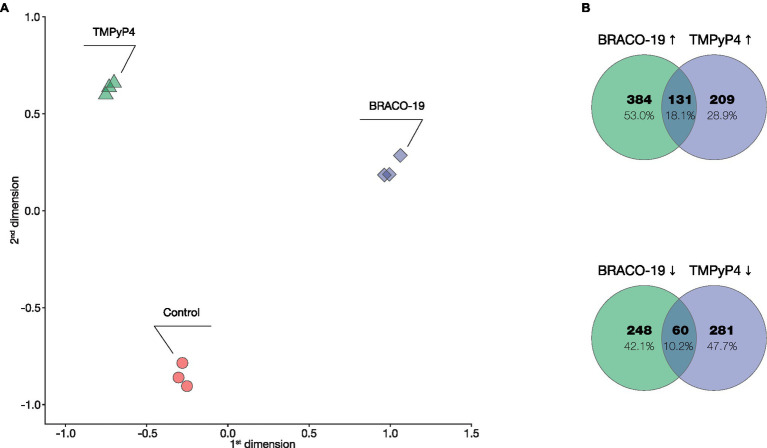
Transcriptional differences between BRACO-19 and TMPyP4 treated *Mycobacterium smegmatis* samples. **(A)**, MDS plot of correlation of genes expression levels of three replicate samples (indicated with different colors) across three different conditions; **(B)**, Venn diagrams showing the genes co-regulated by the presence of BRACO-19 and TMPyP4 compounds, as determined by RNA-seq.

Multidimensional scaling (MDS) of normalized RNA-seq data (three biological replicates and three different conditions) showed a clear separation of samples by condition (Control, BRACO-19, and TMPyP4; Figure 1A). Expression significantly changed (FDR < 0.05, log_2_FC > |1|) for 822 (515↑; 307↓) genes in *M. smegmatis* in response to BRACO-19 and for 680 (339↑; 341↓) genes in response to TMPyP4. Among the 191 differentially expressed genes (DEGs) in response to both BRACO-19 and TMPyP4, 131 genes were upregulated, and 60 genes were downregulated (Figure 1B).

### The Effect of Ligands on the Expression of Genes Linked to G4s

Potential G-quadruplex forming sequences (PQSs) in the *M. smegmatis* MC^2^155 genome were identified using G4-iM Grinder. In total, 53 (14/53 were valid for two genes) PQSs were found in intergenic regions, 17 PQSs were located in promoter regions, 267 PQSs were situated in the mRNA-like strand of the 255 genes, and 398 PQSs were located in the template strand of the 360 genes (Supplementary Table S2).

**Figure 2:**
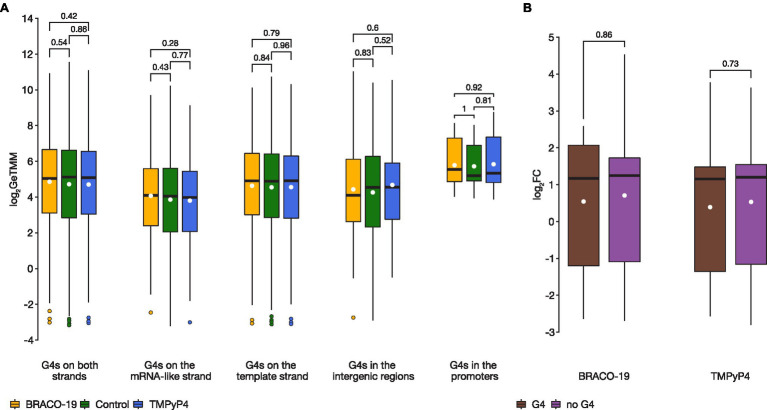
There were no statistically significant differences in expression levels of genes containing G4 motifs between control and treated samples. **(A)**, A boxplot showing expression variance of G4-associated genes between control (green), on exposure to BRACO-19 (yellow), and on exposure to TMPyP4 (blue). The box shows the upper and lower quartiles, and the line within the box shows the median. The white dot indicates the mean. Points above the whiskers indicate outliers. Two-sided Wilcoxon rank-sum test with continuity correction was used to calculate values of *p*; **(B)**, A boxplot showing the change in expression (log_2_FC) of co-regulated genes (*n* = 191) observed by RNA-seq of *M. smegmatis* MC^2^155 exposed to BRACO-19 and TMPyP4 ligands. The box shows the upper and lower quartiles, and the line within the box shows the median. The white dot indicates the mean. Two-sided Wilcoxon rank-sum test with continuity correction was used to calculate values of *p*.

The results of RNA-seq analysis showed that there are no significant differences in expression levels of the bulk of the genes associated with G4s (i.e., those harboring PQSs; Supplementary Figure S2). Further, analyses for PQSs located on mRNA-like strand, template strand, intergenic regions, and in promoters between the control sample and samples exposed to G4 ligands, showed no statistically significant differences (two-sided Wilcoxon test *p* > 0.05; Figure 2A). Besides, there was no difference in genes expression (log_2_FC) relative to G4 strandedness (mRNA-like or template) in intergenic regions, neither in BRACO-19 (Student’s *t*-test *p* = 0.519) nor in TMPyP4 (Student’s *t*-test *p* = 0.645) treated samples. Furthermore, no significant differences were observed in expression levels of genes co-regulated by both ligands, between genes associated with G4s (*n* = 14), and without G4s (*n* = 177; two-sided Wilcoxon test *p* > 0.05; Figure 2B).

To verify our RNA-seq results and G4-stabilizing effect of the ligands, we investigated 10 high-scoring PQS fragments of randomly selected genes that showed: enhanced expression levels in response to ligand treatment (*n* = 4), no significant changes (*n* = 2), or decreased expression levels (*n* = 4) (Supplementary Table S3). The effect of G4-ligands on PQSs stability was determined using thermal denaturation monitored by circular dichroism spectroscopy (Supplementary Figure S3). In the presence of BRACO-19 and TMPyP4, G4 stabilization was observed, nonetheless these results did not correlate with the changes in corresponding genes expression levels.

### Changes in the Expression of DNA Replication and Repair Systems Mediated by G4 Ligands

Additionally, the genes of the replication and repair system were considered (Supplementary Table S4), since G4 stabilizing ligands can damage DNA and cause genome instability (Saranathan and Vivekanandan, 2019). In total, 13 and four genes exhibited more than 2-fold expression level changes under the action of BRACO-19 and TMPyP4, respectively (Table 1).

**Table 1:** Differentially expressed genes of the DNA replication and repair systems in *M. smegmatis* exposed to BRACO-19 and TMPyP4.

Locus tag	*M. tuberculosis* H37Rv homologue	Gene name	Gene product	BRACO-19		TMPyP4	
				log_2_FC	FDR	log_2_FC	FDR
MSMEG_4925	Rv1317c	*alkA*	Transcriptional regulator, Ada family protein/DNA-3-methyladenine glycosylase II	**3.15**	**9.23E-13**	0.62	**0.000607**
MSMEG_4928	Rv1316c	*ogt*	Possible 3-methyladenine DNA glycosylase Mpg	**3.31**	**9.49E-13**	0.44	**0.013258**
MSMEG_5422	Rv1021	*mazG*	Nucleoside triphosphate pyrophosphohydrolase	0.66	**6.09E-06**	**−1.13**	**2.6E-08**
MSMEG_1633	Rv3370c	*dnaE2*	Error-prone DNA polymerase	**1.34**	**1.71E-08**	0.90	**3.88E-06**
MSMEG_3172	Rv1537	*dinB1*	DNA polymerase IV	**1.20**	**1.97E-07**	0.18	0.259968
MSMEG_6443		*dinB3*	DNA polymerase IV	**1.52**	**1.96E-07**	0.33	0.104471
MSMEG_2442	Rv2902c	*rnhB*	Ribonuclease HII	**1.098**	**6.33E-08**	0.45	**0.001482**
MSMEG_6896	Rv0054	*ssbA*	Single-stranded DNA-binding protein	**−1.43**	**3.92E-09**	−0.39	**0.003882**
MSMEG_4701	Rv2478c	*ssbB*	Hypothetical protein	0.93	**0.00116**	**1.24**	**8.09E-05**
MSMEG_1327	Rv0630c	*recB*	Exodeoxyribonuclease V subunit beta	0.31	0.020787	**−1.16**	**2.72E-07**
MSMEG_5397		*recQ*	ATP-dependent DNA helicase RecQ	**2.15**	**0.00002**	**1.54**	**0.0044**
MSMEG_0006	Rv0006	*gyrA*	DNA gyrase subunit A	**1.21**	**1.85E-09**	−0.87	**1.37E-07**
MSMEG_0005	Rv0005	*gyrB*	DNA gyrase subunit B	**1.20**	**8.75E-10**	−0.76	**3.44E-07**
MSMEG_1620	Rv3395c	*imuA*	Hypothetical protein	**1.52**	**0.0006**	−0.12	0.825962
MSMEG_1622	Rv3394c	*imuB*	DNA repair polymerase	**1.28**	**0.000145**	0.96	**0.002957**
MSMEG_5397			ATP-dependent DNA helicase RecQ	**2.15**	**0.000105**	**1.54**	**0.004401**
MSMEG_3885	Rv2092c	*helY*	DEAD/DEAH box helicase	**1.77**	**3.26E-11**	−0.17	0.113436

*Values in bold meet the corresponding selection criteria (FDR < 0.05, log_2_FC > |1|)*.

Genes encoding DNA polymerases DinB and DnaE2, assessor proteins ImuA and ImuB, DNA gyrases GyrA and GyrB, proteins of alkylation repair system AlkA and Ogt, as well as HelY and RecQ helicases were overexpressed on exposure to BRACO-19. Most of these genes are required for translesion DNA synthesis. Polymerase DnaE2 (C-family probable translesion polymerase) is reportedly involved in adaptive mutagenesis in response to DNA damage (Boshoff et al., 2003). Accessory proteins ImuA and ImuB facilitate DnaE2-dependent translesion synthesis^43^, thus contributing to bacterial SOS response. Although the role of DinB1, DinB2 [duplicated in *M. smegmatis*; MSMEG_2294/MSMEG_1014 (both genes had zero total read counts)], and DinB3 (specific for *M. smegmatis*) Y-family polymerases is still not fully understood, it has been reported that DinB1 and DinB3 are typical DNA-dependent DNA polymerases, while DinB2 misincorporates deoxyribonucleotides and ribonucleotides during templated synthesis and lesion bypass (Ordonez and Shuman, 2014). Notably, according to previous reports, neither *dinB1*- nor *dinB2*-encoded PolIV homolog is up-regulated during the mycobacterial damage response (Rand et al., 2003). The up-regulated DNA gyrases (type II DNA topoisomerases) contribute to DNA synthesis by catalyzing the introduction of negative supercoiled turns in an ATP-dependent manner, while double-stranded DNA is unwound by lengthening the RNA polymerase or helicase in front of the progressive replication fork (Aubry et al., 2006). Among genes responsible for alkylation repair, *ogt* and *alkA* had the highest expression levels. Methyltransferase Ogt and AlkA DNA-glycosylase are the major proteins of repair systems; they counteract DNA alkylation damage, thus preventing cell death (Yang et al., 2011).

In TMPyP4-treated *M. smegmatis*, *ssbB* (MSMEG_4701), which encodes the RecA accessory factor required for recombination repair during stress (Singh, 2018), was the only up-regulated repair system gene (log_2_FC = 1.24), and this gene was also upregulated in BRACO-19 case (log_2_FC = 0.93). On the contrary, *ssbA* (MSMEG_6896), which encodes another RecA cofactor (Singh, 2018), was downregulated for both ligands (log_2_FC = −1.43 for BRACO-19, and log_2_FC = −0.3 for TMPyP4). Such a multi-directional change in the level of expression of the aforementioned genes has been described previously for ultraviolet and hypoxia-induced stress in *M. smegmatis* (Singh et al., 2018).

Additional analysis of previously described helicases (DinG, UvrD1, and UvrD2; Thakur et al., 2014; Saha et al., 2019), responsible for the unwinding of the G4-quadruplexes, showed slightly increased expression levels, although only significant (FDR < 0.05) for BRACO-19 (Supplementary Table S1). Besides, DNA helicase RecQ was up-regulated under BRACO-19 and TMPyP4. RNA-activated ATPase/dATPase and 3′-5′ helicase *helY* gene was upregulated on exposure to BRACO-19. In the TMPyP4 case, the ATP-dependent RNA helicase MSMEG_1540 was downregulated (log_2_FC = −2.08).

### Functions and Pathways of *Mycobacterium smegmatis* MC^2^155 Genes Responding to Treatment With G4 Ligands

Since there was no strong evidence that BRACO-19 and TMPyP4 ligands affect the expression of G4-carrying genes in *M. smegmatis*, further analysis of transcriptomic data was performed. Differentially expressed genes were subjected to functional classification and pathway enrichment analysis based on the Kyoto Encyclopaedia of Genes and Genomes (KEGG) and gene ontology (GO) databases (Supplementary Table S5).

**Figure 3:**
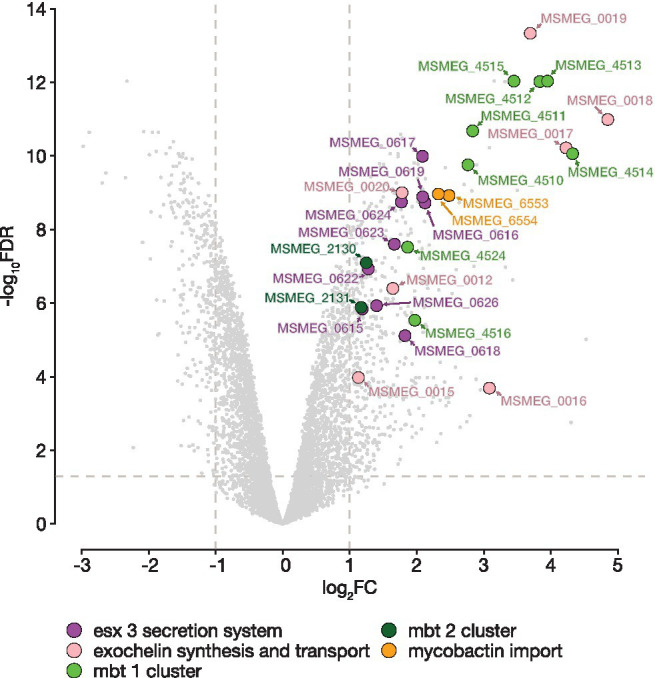
BRACO-19 compound affects *M. smegmatis* MC^2^155 iron metabolism. Volcano plot indicates the distribution of DEGs for the *M. smegmatis* MC^2^155 exposed to BRACO-19 ligand. Log transformed fold changes (log_2_FC) are plotted on the *x*-axis, and significance (−log_10_FDR) is plotted on the *y*-axis. Colors denote genes involved in iron metabolism with a significantly increased level of expression (FDR < 0.05, log_2_FC > |1|).

For BRACO-19, only one KEGG pathway—msm01053 “Biosynthesis of siderophore group nonribosomal peptides” (*p*_adj._ = 1.28e-05), was enriched for upregulated genes. This pathway includes *mbt* cluster genes (Figure 3; Supplementary Table S6), which are responsible for the synthesis of the most important iron-chelating compounds—siderophores (mycobactin and carboxymycobactin in mycobacteria; Sritharan, 2016). Analysis of the exochelin locus, which is a specific siderophore for non-virulent mycobacteria, revealed similar changes. Also, overexpression of genes involved in siderophore transport and acquisition was observed. Among these genes were the iron-dependent transporters *irtAB* and ESX-3 operon. The ATP binding cassette transporter IrtAB imports iron-bound siderophores across the inner membrane, while the type VII secretion system Esx-3 plays a crucial role in iron acquisition through secretion of a pair of PE–PPE family proteins. In turn, iron storage genes *bfrA* and *bfrB*, encoding a heme-containing bacterioferritin (BfrA) and a ferritin-like protein (BfrB), were slightly downregulated. It is worth noting that almost all the aforementioned genes are controlled by the IdeR (iron-dependent regulator; MSMEG_2750; Sritharan, 2016). The *ideR* gene was slightly upregulated (log_2_FC = 0.65) compared to the control, notably, there is a high-scored (*x* = 57) quadruplex with four G-tetrads (**GGGG**AT**GGGG**TTGCCGAAC**GGGG**AGGTGGT**GGGG**) located on the template strand of the gene.

In the case of TMPyP4-exposed bacteria, the following biological process GO terms were enriched: GO:0006351 “transcription, DNA-templated” (*p*_adj._ = 0.002) for upregulated genes, and GO:0006526 “arginine biosynthetic process” (*p*_adj._ = 1.21e-05) for downregulated genes. The KEGG pathways associated with upregulated genes in the TMPyP4-treated sample, were msm00920 “Sulfur metabolism” (*p*_adj._ = 0.02), and msm02010 “ABC transporters” (*p*_adj._ = 0.02), and one pathway associated with down-regulated genes was msm00220 “Arginine biosynthesis” (*p*_adj._ = 0.03). Primarily, GO:006351 term included potential transcription factor genes with increased expression that may reflect significant changes in metabolism in response to ligand action. KEGG pathway msm02010 included almost all the genes in msm00920 pathway (7/8) and consisted of the genes responsible for sugar transport, phosphates, and sulfur-containing amino acids. At the same time, genes involved in the synthesis of the amino acid arginine (MSMEG_3046, MSMEG_3047, MSMEG_3769-MSMEG_3776) were downregulated in TMPyP4-treated *M. smegmatis*. Of these, carbamoyl phosphate synthetases units A (MSMEG_3046) and B (MSMEG_3047) are involved in pyrimidine and arginine biosynthesis, catalyzing the conversion of glutamine to carbamoyl phosphate, while products of the *argDBJC* (MSMEG_3773-MSMEG_3776) genes are part of a five-enzyme pathway that uses glutamate to produce ornithine. Both products are used in the urea cycle to produce arginine under the action of ArgG (MSMEG_3770) in conjunction with ArgF (MSMEG_3772) and ArgH (MSMEG_3769) enzymes.

## Discussion

Here, we employed RNA-seq analysis to demonstrate the impact of G4-ligands, BRACO-19 and TMPyP4, on *M. smegmatis* transcription and elucidate their mechanism of action. *Mycobacterium smegmatis* was used in this study because it is considered as a valuable model organism for mycobacterium study due to the close association with *M. tuberculosis* regarding biochemical properties and genetic information (Joseph Antony Sundarsingh et al., 2020), and, importantly, has a similar GC composition. Both ligands used are best known as stabilizers of human telomeric G4s, which implies cancer-specific antiproliferative activity (Rha et al., 2000; Incles et al., 2004). However, they also exhibit broad-spectrum G4 stabilizing activity and, along with other pan-quadruplex ligands, are being tested for their therapeutic potential against various bacterial species, including *M. tuberculosis*. In the case of *M. smegmatis* (this study) and *M. tuberculosis* (Beaudoin et al., 2014; Mishra et al., 2019), both compounds inhibited the growth at submicromolar concentrations, but TMPyP4 was slightly more active for *M. smegmatis*.

Both BRACO-19 and TMPyP4 have been reported to stabilize synthetic G4s from *M. tuberculosis*. TMPyP4 has also been shown to reduce transcription of PQS-containing genes (Beaudoin et al., 2014; Mishra et al., 2019). Based on our findings, there was no strong evidence of significant up- or down-regulation of genes associated with PQSs caused by G4-ligands. Notably, we analyzed ligands’ effects both separately and in conjunction to account for specific DEGs and the co-regulated ones, respectively. However, even for co-regulated genes, we were unable to find concrete evidence of ligand-mediated G4-stabilization. The examples of DEGs that are co-regulated by both compounds and harbor/neighbor PQSs include MSMEG_4727↓, MSMEG_1680↓, MSMEG_4689↓, MSMEG_3948↓, MSMEG_2242↑, MSMEG_3121↑, and MSMEG_2148↑ (contain PQSs on the template strand of the gene); MSMEG_1488↑, MSMEG_0868↑, MSMEG_6223↑, MSMEG_5397↑, and MSMEG_4202↑ (contain PQSs on the mRNA-like strand); MSMEG_6567↑ (contains one PQS on each strand); MSMEG_3917↑ (contains a PQS on the template strand in the intergenic region), and MSMEG_4643↓ (contains a PQS on the mRNA-like strand in the intergenic region). Moreover, additional CD assays showed stabilization of PQSs by BRACO-19 and TMPyP4, which did not correlate with the results of transcriptome analysis.

We hypothesize that the ligands act primarily during replication, when the majority of G4s are formed (as has been proven for eukaryotic cells; Rodriguez et al., 2012), and transcription changes stem partly from the disruption of replication (Lerner and Sale, 2019). It is possible that the observed transcription changes illustrate compensatory response rather than primary ligands’ effects. G4 stabilization upon replication may cause polymerase stalling and SOS-response (Maslowska et al., 2019). Enhanced recruitment of the translesion synthesis machinery or timely ligand dislodging and G4 unwinding by helicases would then be required for the tolerance of ligand-induced toxicity. In the present study, under the action of BRACO-19, we identified modest upregulation of DnaE2, DinB1, and DinB3 translesion polymerases; however, no significant changes in expression levels of SOS-regulation genes (*recA* and *lexA*) were registered. Meanwhile, treatment with BRACO-19 and TMPyP4 resulted in overexpression of *M. smegmatis* specific gene MSMEG_5397 encoding RecQ helicase (Table 1). The importance of RecQ DNA helicases in G4 unwinding in bacteria has been previously demonstrated in the case of *Escherichia coli*, although RecQ protein in *M. smegmatis* is not homologous to *E. coli* (Huber et al., 2006; Singh, 2017). In this respect, it is not possible to affirm the relationship between the overexpression of the MSMEG_5397 and the action of ligands. In addition, BRACO-19 increased the expression of the *helY* gene. In *M. smegmatis* HelY unwinds 3′-tailed RNA duplexes and RNA–DNA hybrids, but is unable to unwind a 3′-tailed duplex in which the loading strand is DNA. It is possible that HelY participates in the unwinding of RNA G4 quadruples; however, the eukaryotic homologs Ski2 and Mtr4 do not possess such properties (Uson et al., 2015).

BRACO-19-induced overexpression of iron metabolism-related genes may be an additional indication of the increased need for metalloproteins involved in replication and DNA repair (Expert et al., 2008; Puig et al., 2017), i.e., the Fe/S cluster-containing enzymes (Puig et al., 2017). Interestingly, similar expression patterns for exochelin biosynthesis, exochelin uptake, and mycobactin synthesis genes have been reported for *M. smegmatis* grown in iron deficiency conditions (Ojha and Hatfull, 2007) and under both oxidative and nitrosative stress (Namouchi et al., 2016).

The observed changes in iron metabolism in response to BRACO-19 treatment may, obviously, have a more straightforward explanation. They could all be driven by a single BRACO-19-sensitive (G4-harboring) master gene. We have considered such a possibility, and the iron-dependent regulator (*ideR*) was a primary candidate. However, *ideR* exhibited only minor expression changes and only in BRACO-19-treated samples. This finding goes against the straightforward explanation. Instead of primary effects, we are probably witnessing an adaptation to G4-related general replication stress.

It is reasonable to assume that the global replication stress arises from direct ligand interactions with G4 DNA. Interactions with G4 RNA have not been considered herein but should probably be the subject of future studies. Persistent mRNA G4s are expected to impair translation and tend to be depleted in prokaryotes (Guo and Bartel, 2016). Recent studies advocate the existence of transient G4s in both eukaryotic and prokaryotic RNA (Kwok et al., 2018; Shao et al., 2020). Importantly, G4 folding modulates the function of non-coding RNA (ncRNA), and stabilization of the ncRNA G4s by the ligands may eventually affect the transcription profile (Shao et al., 2020). Thus, a combination of G4-seq and rG4-seq (Marsico et al., 2019) may prove helpful to further elucidate the key targets of G4 ligands and the mechanisms behind their antibacterial activity.

Notably, nucleoid proteins, transcription factors, and probably some other factors affect G4 stability *in vivo* (Parekh et al., 2019), arguing that chromatin immunoprecipitation assays with G4-specific antibodies (G4-ChIP-seq) are needed to *bona fide* confirm G4 formation. The actual number of G4 targets may be substantially lower than that predicted by bioinformatics tools, which is the case in eukaryotes (Hänsel-Hertsch et al., 2018). Moreover, regarding the transient nature of G4 structures (Yadav et al., 2021), the effects of the ligands should ideally be monitored in dynamics. We only analyzed a single time point and emphasize that the presented results, although valid for selected conditions, are insufficient for comprehensive characterization of the G4 ligands.

In conclusion, we provided a transcriptome-wide analysis of *M. smegmatis* under the action of two G4 ligands. Although there have been significant changes in the transcriptional profile of the bacterium, neither BRACO-19 nor TMPyP4 treatment induced a significant change in G4 associated genes. In turn, for the BRACO-19 compound, we found significant changes in the replication and repair system genes, which is, undoubtedly, evidence of the induced stress. Changes in iron metabolism genes also support this hypothesis. For the TMPyP4 compound, we did not find changes in the repair and replication system, however, significant changes in transcription factors and the arginine biosynthesis system may indicate multiple biological targets for this compound, which does not eliminate direct interaction with DNA.

## Data Availability Statement

The datasets presented in this study can be found in online repositories. The names of the repository/repositories and accession number(s) can be found in the article/Supplementary Material.

## Author Contributions

ES, MZ, DB, and AV wrote the main manuscript text. DB, JB, KK, VV, and IB prepared figures and tables. MM, TV, MZ, and IB cultivated mycobacteria and performed susceptibility tests. DB, KK, and VV conducted RNA-seq analysis. ES, EI, MM, JB, and AV designed the experiment. All authors reviewed the manuscript. All authors contributed to the article and approved the submitted version.

## Funding

This research was funded by the Russian Science Foundation (RSF, grant number 19-75-10109).

## Conflict of Interest

The authors declare that the research was conducted in the absence of any commercial or financial relationships that could be construed as a potential conflict of interest.

## Publisher’s Note

All claims expressed in this article are solely those of the authors and do not necessarily represent those of their affiliated organizations, or those of the publisher, the editors and the reviewers. Any product that may be evaluated in this article, or claim that may be made by its manufacturer, is not guaranteed or endorsed by the publisher.

## Footnotes

^1^ https://www.ncbi.nlm.nih.gov/sra

^2^ https://ftp.ncbi.nih.gov/pub/COG/COG2020/data/
