# Supplementary material for: Genome-Wide Transcriptional Response of Mycobacterium smegmatis MC2155 to G-Quadruplex Ligands BRACO-19 and TMPyP4
Source: Front Microbiol. 2022 Mar 4;13:817024. doi: 10.3389/fmicb.2022.817024 (PMC8931766; doi:10.3389/fmicb.2022.817024)
Supplement: Supplementary file 7 [file Data_Sheet_1.PDF]

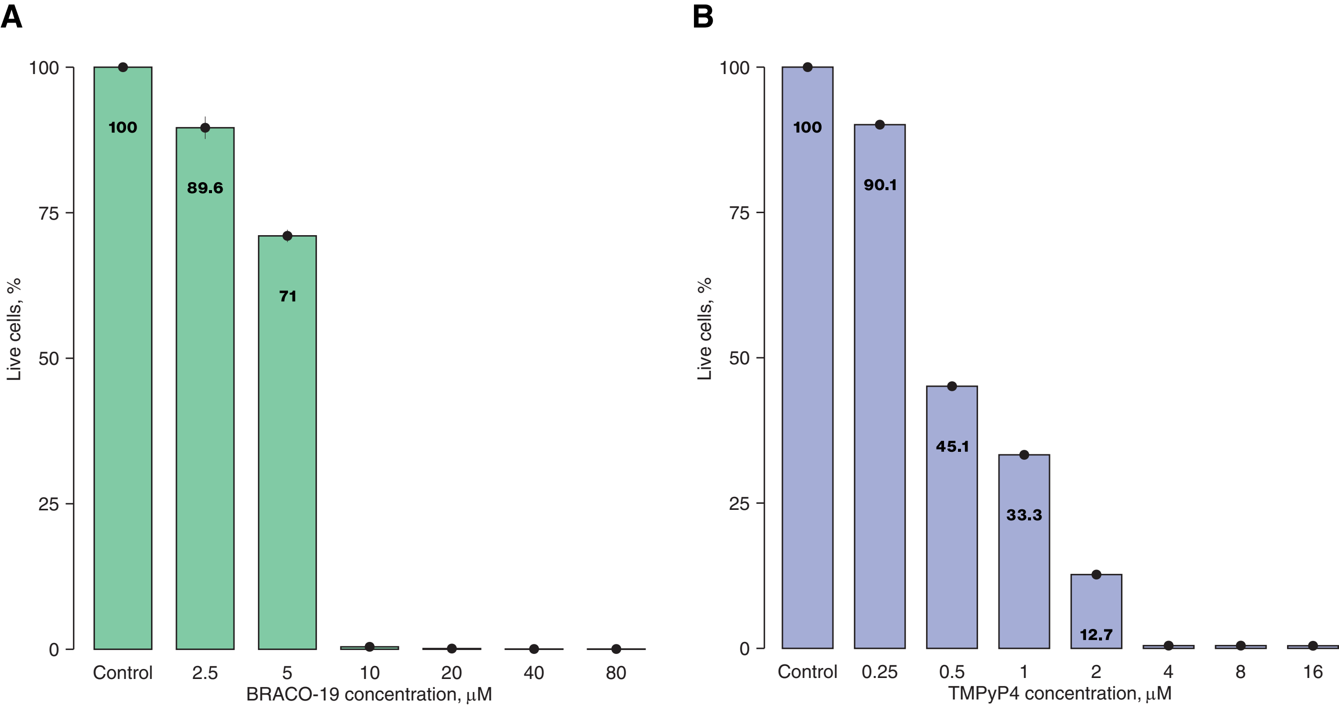

Figure S1. G4 ligands induced *M. smegmatis* growth Inhibition. Antimycobacterial activity of **A**, BRACO-19 and **B**, TMPyP4 against *M. smegmatis* MC^{2}155.
