# Supplementary material for: Genome-Wide Transcriptional Response of Mycobacterium smegmatis MC2155 to G-Quadruplex Ligands BRACO-19 and TMPyP4
Source: Front Microbiol. 2022 Mar 4;13:817024. doi: 10.3389/fmicb.2022.817024 (PMC8931766; doi:10.3389/fmicb.2022.817024)
Supplement: Supplementary file 8 [file Data_Sheet_2.PDF]

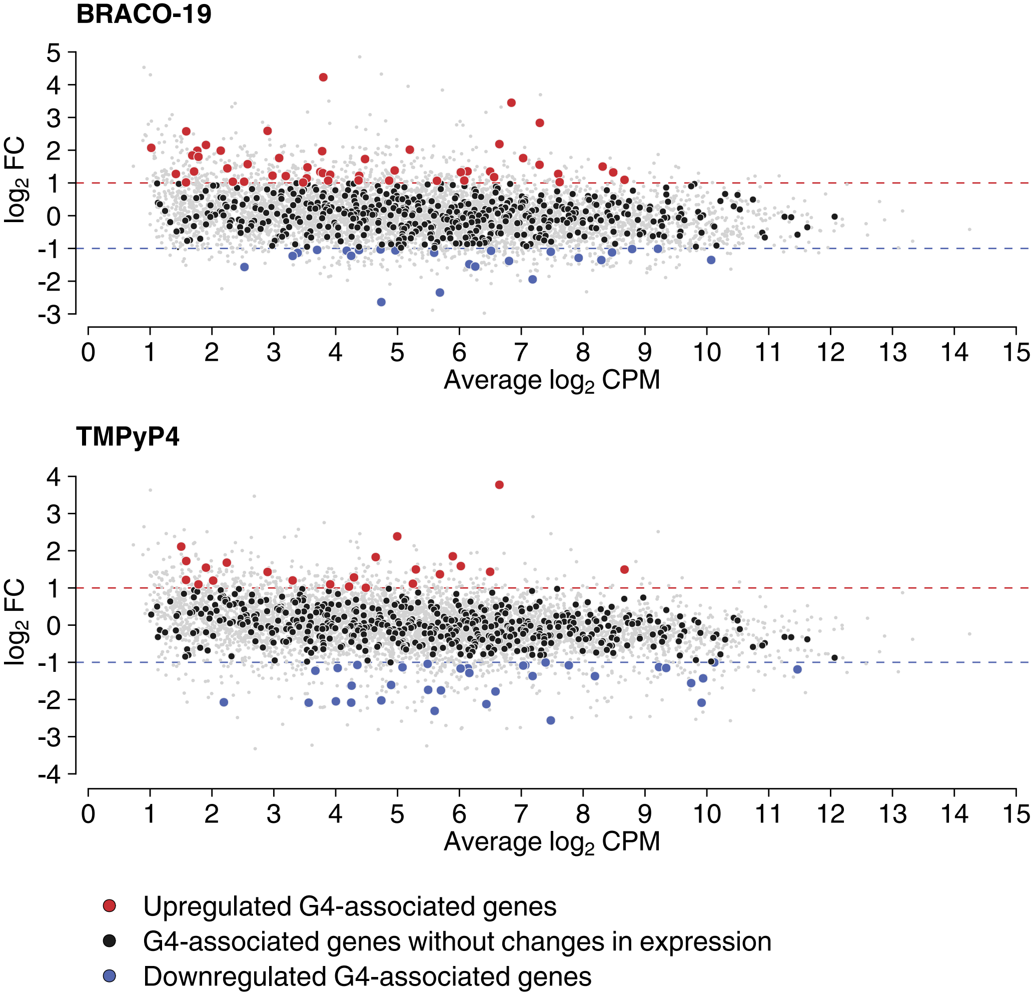

Figure S2. Mean difference plot showing the log-fold change and average abundance of each G4-associated gene.
