# Supplementary material for: Genome-Wide Transcriptional Response of Mycobacterium smegmatis MC2155 to G-Quadruplex Ligands BRACO-19 and TMPyP4
Source: Front Microbiol. 2022 Mar 4;13:817024. doi: 10.3389/fmicb.2022.817024 (PMC8931766; doi:10.3389/fmicb.2022.817024)
Supplement: Supplementary file 9 [file Data_Sheet_3.PDF]

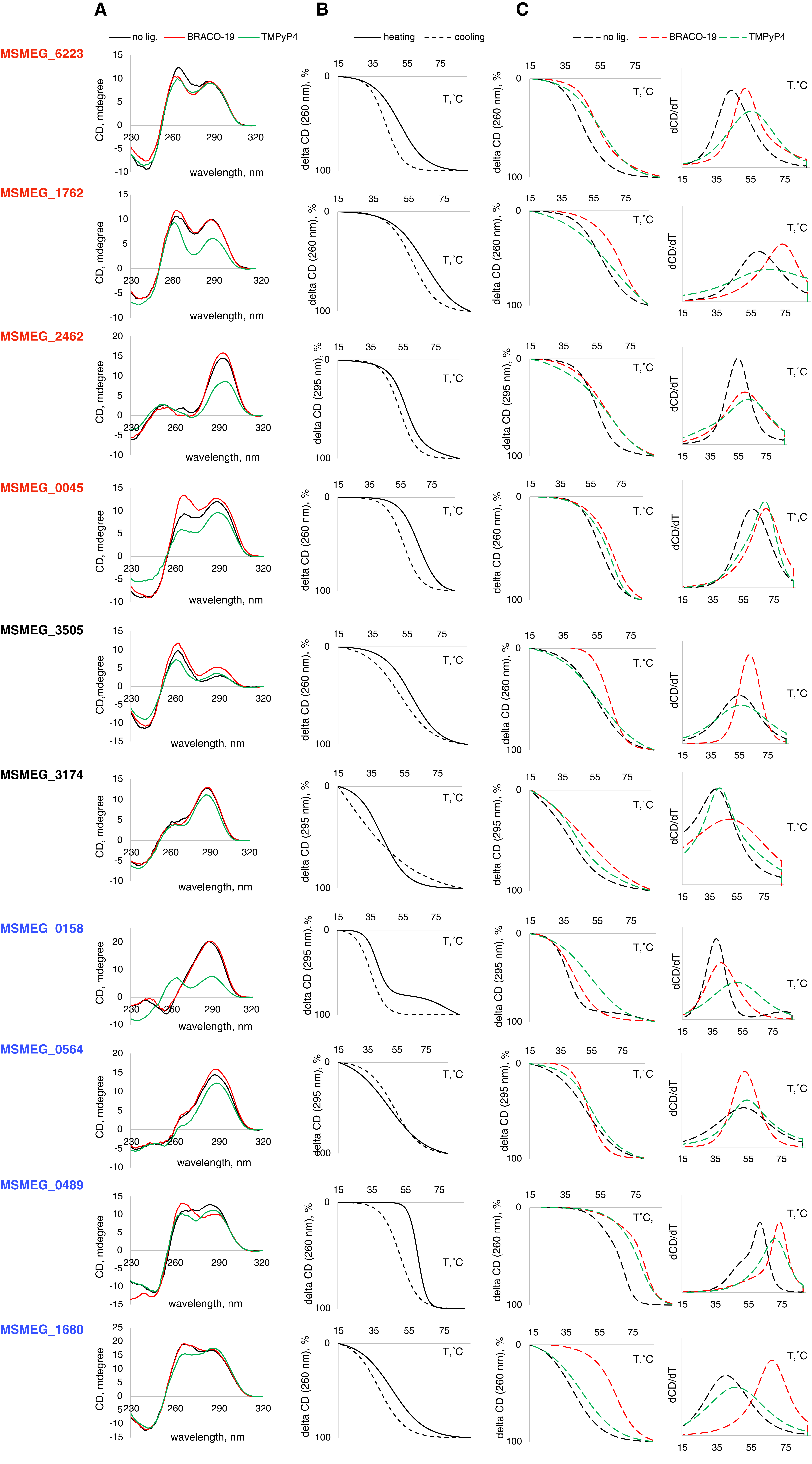

Figure S3. Circular Dichroism Spectra analysis. **A**, circular dichroism spectra; **B**, hysteresis (melting and annealing curves); **C**, Ligand-induced stabilization(average of the annealing and melting curves obtained in the presence or in the absence of the ligands). Red locus tags show G4s from genes that showed enhanced transcription upon treatment with the ligands; Black locus tags show G4s from genes tthat showed no transcription changes upon treatment with the ligands; Blue locus tags show G4s from genes that showed decreased transcription upon treatment with the ligands.
